# Effect of important modifiers on harmful effects in evidence synthesis practice of adverse events were insufficiently investigated: an empirical investigation

**DOI:** 10.1186/s12874-023-01928-2

**Published:** 2023-04-28

**Authors:** Xiaoqin Zhou, Xi Yang, Fei Cai, Li Wang, Chang Xu, Pengli Jia

**Affiliations:** 1 Center of Biostatistics, Design, Measurement and Evaluation (CBDME), Department of Clinical Research Management, West China Hospital, Sichuan University, Chengdu, Sichuan China; 2 Research Center of Clinical Epidemiology and Evidence-Based Medicine, West China Hospital, Sichuan University, Chengdu, Sichuan China; 3 Key Laboratory for Population Health Across-Life Cycle, Ministry of Education, Anhui, China; 4 School of Public Health, Anhui Medical University, Anhui, China; 5 School of Management, Shanxi Medical University, No.56, Xinjian South Road, Yingze District, Taiyuan, 030001 Shanxi China

**Keywords:** Meta-analysis of adverse events, Harmful effect, Effect modifier, Empirical investigation, Methodological guideline

## Abstract

**Background:**

Safety is important in the assessment of health interventions, while the results of adverse events are often susceptive to potential effect modifiers since the event risk tends to be rare. In this study, we investigated whether the potential impact of the important effect modifiers on harmful effects was analyzed in meta-analyses of adverse events.

**Methods:**

Systematic reviews of healthcare interventions, had adverse events as the exclusive outcomes, had at least one meta-analysis, and published between 1^st^ January 2015, and 1^st^ January 2020 were collected. An adverse event was defined as any untoward medical occurrence in a patient or subject in healthcare practice. Six effect modifiers that are the most important for harmful effects were identified by a group discussion. The proportions of eligible systematic reviews that investigated the potential impact of the six effect modifiers on harmful effects were summarized.

**Results:**

We identified 279 systematic reviews eligible for this study. Except for the modifier of interventions/controls (70.61%, 197/279), most of the systematic reviews failed to investigate the potential impact of treatment duration (21.15%, 59/279), dosage (24.73%, 69/279), age (11.47%, 32/279), risk of bias (6.45%, 18/279), and source of funding (1.08%, 3/279) on harmful effects. Systematic reviews with meta-analyses containing more studies were more likely to investigate the potential impacts of these modifiers on the effects, but the proportion was still low (2.3% to 33.3%). Systematic reviews that developed a protocol were significantly more likely to investigate the potential impact of all these effect modifiers (e.g. treatment duration: odds ratio = 5.08, 95% CI: 2.76 to 9.35) on the results.

**Conclusions:**

Current systematic reviews rarely investigated the potential impact of the important effect modifiers on harmful effects. Methodological guidelines for meta-analysis of adverse events should consider “effect modifier” as one of the domains to help systematic review authors better investigate harmful effects.

**Supplementary Information:**

The online version contains supplementary material available at 10.1186/s12874-023-01928-2.

## Introduction

Safety is as important as efficacy in the assessment of health interventions. As recommended by the latest Cochrane handbook (version 6.2), all systematic reviews of interventions should investigate the adverse effects of interventions [1]. Adverse events pose substantial challenges for statistical modeling and inference in individual trials as well as in meta-analyses; a particular challenge is the zero-event problem because the event risk generally tends to be low [2–5]. Due to the same reason, the results of adverse events are often susceptive to potential effect modifiers, such as treatment duration, doses of interventions, population characteristics (e.g., age, weight), bias in the study design, implementation, and reporting [6–8].

Assessing the potential impact of these modifiers on harmful effects in a systematic review and meta-analysis could provide further information for healthcare decision-making. For example, the assessment could include whether a higher dose or a longer treatment duration would lead to a higher risk of adverse events, whether lack of blinding or selective reporting on adverse events moves harmful effect estimates toward the null, or whether different age groups have different event risks. Taking into account the potential impact of these modifiers could provide us with more reliable evidence for decision-making. Therefore, in addition to appropriately dealing with the zero-event problem [9], investigating the potential impact of effect modifiers on the results should also be a routine process in a systematic review and meta-analysis assessing adverse events.

Several well-established and easy-to-implement methods (e.g., subgroup analysis, sensitivity analysis) can be used to investigate the impact of these modifiers on the results [10, 11]. Unfortunately, current guidelines (e.g. [1, 12–14].) for meta-analysis seldom highlight the importance of addressing the impact of the effect modifiers on the effects for adverse events, because current methodological guidelines mainly focus on the efficacy of an intervention rather than the harm. Developing evidence-based methodological guidelines, specifically for a meta-analysis of adverse events would largely promote evidence synthesis practice for harms assessment. One important step for the development of such guidelines is to understand how harmful effects were investigated in meta-analyses of adverse events.

We have investigated how zero-event studies were dealt with in meta-analyses of adverse events recently [15]. In this article, we further investigate the analysis of the impact of effect modifiers on the results in these meta-analyses.

## Methods

The current study is an extension of a recent empirical investigation about methods to deal with zero events by our group [15]. To further explore the potential impact of the aforementioned effect modifiers on harmful effects, a new protocol was developed and changes in the protocol were recorded (see Additional file 1). We reported the current study according to the PRIO-harms checklist [16].

### Data source

We used the dataset collected in 2020 through PubMed, which consists of 511 systematic reviews of healthcare interventions in humans, published between 1^st^ January 2015, and 1^st^ January 2020. These reviews had adverse events as the exclusive outcomes, and each review had at least one meta-analysis [15]. The primary search strategy and literature search were conducted by an information scientist, and have been documented elsewhere [15]. The primary literature screen was conducted through Rayyan (https://rayyan.qcri.org/) by two participants independently and again with the details were documented in our previous studies [15]. We defined adverse events as any untoward medical occurrence in a patient or subject in healthcare practice [17].

### Selection of meta-analyses

Systematic reviews of incidence proportions were not considered in the current study since such types of systematic reviews only assessed the baseline risks instead of harmful effects. In addition, surgical, device, radiation oncology, or complementary interventions were also excluded since treatment duration, doses, or some domains of risk of bias (e.g., blinding) are not applicable [18]; we therefore only focused on those systematic reviews with interventions of drugs or biologics. Considering that clinical trials are the main source of high-quality evidence for safety assessment, this study was restricted to systematic reviews of clinical trials. Two authors (FC and LW) screened the full text of the 511 systematic reviews independently and any controversy was dealt with by discussion.

### Data collection

Data collection was conducted by the lead author (XQ) and then double-checked by another author (XY) using Excel (Microsoft, USA). Any discrepancies were discussed and resolved through consensus. The following characteristics of the systematic reviews were extracted: name of the first author, year of publication, region of the corresponding author, type of meta-analysis (pairwise vs. network meta-analysis), the total number of trials included, number of outcomes examined, number of trials for each outcome, the topic of the systematic review, publication of protocol, and reporting of funding information.

### Identification of effect modifiers

We pre-defined six effect modifiers that are the most important for harmful effects based on online group discussion (CX, XQ, LF, LFK) and further consultants from one pharmacist (JX), one clinician on cardiovascular disease, and one methodologist on study design. These effect modifiers were identified from “Participants”, “Intervention”, “Comparison”, “Outcome”, and “Study design” (PICOS) [19] of each study for a meta-analysis. Finally, the effect modifiers we considered including different interventions/controls, treatment duration, dosage, population characteristics (e.g., age), risk of bias, and source of funding. Here different interventions/controls means the interventions or controls differs across included studies. For example, some studies used Placebo as control while some used active treatment as control. In some meta-analyses, studies comparing multiple treatments (200 mg Drug A plus 15 mg Drug B) with the add-on treatment (15 mg Drug B plus placebo) were simply regarded as the net effect of Drug A (i.e., Drug A vs. placebo); However, the add-on treatment would also cause adverse events that impact the harmful effects [20]. Therefore, in the current study, we treat studies with “Drug A plus Drug B vs. Drug B plus placebo” have different intervention/control to studies with “Drug A vs. Placebo”. We did not differentiate between “treatment duration” and “follow-up”; the latter generally refers to a longer period [21]. For population characteristics, there are many that would impact the effects, including age, gender, medical condition, special population with high risk, etc., while age is the most commonly reported information in published systematic reviews, and we selected it as a representative. In addition, a seventh item, whether the authors ranked the confidence of the evidence of harmful effects, was also collected.

In some cases, a question was not applicable to certain systematic reviews, and it was assigned as “NA”. For example, some systematic reviews with included studies used the same intervention and control; then, it was impossible to investigate the impact of different interventions and controls on harmful effects. For simplicity, the same drug with different dosages was treated as the same intervention. For risk of bias, different types of instruments might be used in systematic reviews; therefore, we recorded the detailed domains of bias (e.g., blinding) when applicable. We also collected information about the methods utilized to investigate the impacts of these modifiers on the results.

### Statistical analysis

The primary outcome of this study was the proportion of eligible systematic reviews that investigated the potential impact of the six effect modifiers on harmful effects. The secondary outcomes included the proportion of eligible systematic reviews that ranked the level of the evidence on the results, as well as the methods utilized to investigate the impacts of these modifiers on the results.

Sensitivity analysis was employed by excluding systematic reviews with network meta-analyses. Two additional sensitivity analyses were also conducted by limiting the analyses to systematic reviews with meta-analyses containing ≥ 5 and ≥ 10 studies across the outcomes.

Because of the increasing focus on the protocol development for systematic reviews [22], we further compared the odds of the proportions of investigating the impact of the effect modifiers on harmful effects among systematic reviews with a protocol to those without a protocol. We used the odds ratio (OR) to measure the effects because it is a “portable” effect estimate [23]. There were no zero events occurring in the comparisons; therefore, we did not need to specify methods to deal with zero-events.

All statistical analyses were performed by Excel (Microsoft, USA) and MetaXL (version 5.3, EpiGear, Australia). The significance level was pre-specified as alpha = 0.05.

## Results

### Characteristics of the systematic reviews

Consequently, 279 systematic reviews (61.2% of our original dataset of 456 systematic reviews) were eligible (see Additional file 2 and Additional file 6).

Table 1 presents the characteristics of the 279 systematic reviews on adverse events. Research groups from Asia contributed the most (41.94%) of the systematic reviews, followed by European (29.39%) and American (22.22%) groups. There were 241 (86.38%) systematic reviews that conducted pairwise meta-analyses and 38 (13.62%) that conducted network meta-analyses. The median number of trials included was 16 (IQR: 10 to 32), and the majority (75.27%) of the systematic reviews included contained 10 or more trials. A protocol was developed by 77 (27.60%) of the systematic reviews, and most failed to develop or report a protocol (72.40%). In terms of the topics, cancer (40.50%), diabetes (10.39%), osteoarticular diseases (8.96%), cardiovascular diseases (5.73%), and mental disorders (4.30%) were the most investigated.

**Table 1:** Basic characteristics of included systematic reviews on adverse events by drug or biologics

Basic characteristics	No. of systematic reviews (*N* = 279)
**Region of the corresponding author**	
Africa	14 (5.02%)
America (North and South)	62 (22.22%)
Asia	117 (41.94%)
Europe	82 (29.39%)
Oceania	4 (1.43%)
**Type of meta-analysis**	
Pairwise meta-analysis	241 (86.38%)
Network meta-analysis	38 (13.62%)
**The number of trials included**	16 (IQR: 10 to 32)
1 to 9 (minimum is 3)	69 (24.73%)
10 to 29	135 (48.39%)
30 or more (maximum is 597)	75 (26.88%)
**Protocol**	
Yes	77 (27.60%)
No	202 (72.40%)
**Topic of disease**	
Cancer	113 (40.50%)
Diabetes	29 (10.39%)
Osteoarticular diseases	25 (8.96%)
Cardiovascular diseases	16 (5.73%)
Mental disorders	12 (4.30%)
Inflammatory bowel disease	10 (3.58%)
Respiratory diseases	9 (3.23%)
Blood and lymphatic system diseases	8 (2.87%)
Neuropathy diseases	7 (2.51%)
Coagulation and anticoagulation	6 (2.15%)
HCV/HIV/HPV	6 (2.15%)
Autoimmune diseases	5 (1.79%)
Infection	5 (1.79%)
Neuropathy diseases	5 (1.79%)
Addition	4 (1.43%)
Lower urinary tract symptoms	3 (1.08%)
Inflammation	3 (1.08%)
Others	13 (4.66%)
**Funding**	
No funding	94 (33.69%)
Not reported	85 (30.47%)
Non-profit funding	94 (33.69%)
For profit funding	6 (2.15%)

*HCV* Hepatitis C virus, *HIV* human immunodeficiency virus, *HPV* human papillomavirus

### Investigation of the impact of effect modifiers on harmful effects

**Fig. 1:**
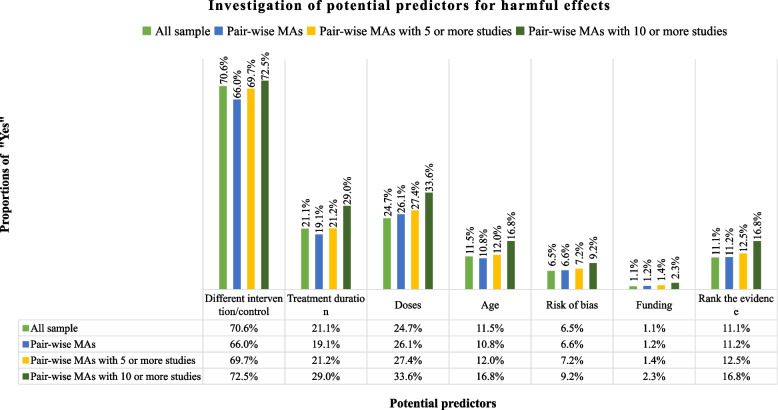
The investigation of the potential impact of effect modifiers on harmful effects for eligible systematic reviews of adverse events

Figure 1 presents the proportions of systematic reviews that investigated the impact of effect modifiers on harmful effects. Generally, most of the systematic reviews investigated the potential impact of different interventions or controls on harmful effects (70.61%, 197/279). However, for the rest effect modifiers, the majority of the systematic reviews failed to investigate the potential impact of them on harmful effects: 21.15% (59/279) of the systematic reviews investigated the impact of treatment duration, 24.73% (69/279) investigated the impact of dosage, 11.47% (32/279) investigated the impact of age, 6.45% (18/279) investigated the impact of risk of bias, and 1.08% (3/279) investigated the impact of source of funding on harmful effects. In addition, only 11.11% (31/279) ranked the evidence of harmful results, all of which used the GRADE (Grading of Recommendations Assessment, Development and Evaluation) approach [24].

For 18 systematic reviews that investigated the impact of the risk of bias on the effects, 9 investigated blinding or not on the effects, 8 investigated the overall quality on the effects, 2 investigated allocation concealment, random sequence generation, and selective reporting on the effects, separately (see Additional file 3).

### Sensitivity analyses

The results of the sensitivity analyses were presented in Fig. 1 and Additional files 4 and 5. After restricting the systematic reviews to those only conducting pairwise meta-analyses, there was a slight decrease in the proportion of investigating different interventions/controls, treatment duration, and age on harmful effects. When we further restricted the systematic reviews to those with meta-analyses containing 5 or more studies, there was a slight increase in all of the 6 domain, in addition to different interventions/controls. In addition, we also observed more systematic reviews ranked the evidence. When further restricted to the systematic reviews with meta-analyses containing 10 or more studies, the increase of the proportion was more obvious.

### Methods used for the investigation

Of the 241 systematic reviews with pairwise meta-analyses, 67.63% (163/241) investigated the impact of at least one of the 6 effect modifiers on harmful effects. Subgroup analysis was the most commonly employed method to investigate the impacts (88.96%, 145/163), followed by meta-regression analysis (12.88%, 21/163) and sensitivity analysis (4.29%, 7/163). We also recorded 5.52% (9/163) that used separate meta-analyses to investigate the impacts. It should be noted that, in addition to the above four methods, we recorded 2 systematic reviews that used the person-time instead of the total event count as a solution to deal with the potential impact of different treatment duration on the effects.

### The role of protocol development

**Fig. 2:**
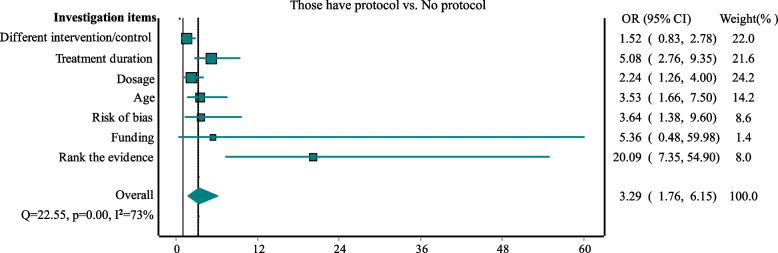
Comparison for systematic reviews with protocol to those without protocol

We compared the 77 systematic reviews that developed a protocol to those 202 that did not develop or report a protocol for investigating the impacts. Figure 2 presents the results, suggesting that those systematic reviews with a protocol were more likely to investigate the potential impact of the effect modifiers on harmful effects: different interventions/controls (OR = 1.52, 95%CI: 0.83 to 2.78), treatment duration (OR = 5.08, 95%CI: 2.76 to 9.35), dosage (OR = 2.24, 95% CI: 1.26 to 4.00), age (OR = 3.53, 95% CI: 1.66 to 7.50), risk of bias (OR = 3.64, 95% CI: 1.38 to 9.60), source of funding (OR = 5.36, 95%CI: 0.48 to 59.98). Moreover, those with a protocol were more likely to rank the grade of evidence for the results (OR = 20.09, 95% CI: 7.35 to 54.90).

## Discussion

In this study, we explored systematic reviews of adverse events for healthcare intervention of drugs or biologics, and examined whether they investigated the potential impact of six important effect modifiers on harmful effects. We found that the majority (67% or more) of these systematic reviews failed to investigate the potential impact of treatment duration, dosage, population characteristics (age), risk of bias, and source of funding on harmful effects. We also found that these systematic reviews seldom ranked the confidence of the results. Our sensitivity analyses suggested that those systematic reviews with meta-analyses containing more studies were more likely to investigate the potential impacts of these modifiers on the effects. However, even for these systematic reviews, the proportion of investigation was still low (2.3% to 33.3%). In addition, systematic reviews that developed a protocol were significantly more likely to investigate the potential impact of effect modifiers on the results.

Among the six pre-defined effect modifiers, treatment duration was one of the most important effect modifiers, which has been highlighted by Böhning et al. [6] Generally, longer treatment duration may lead to more adverse events. For treatment duration, for those systematic reviews that investigated its impact on the effects, researchers tended to address the between-study difference of the treatment duration. This makes sense if the treatment durations are the same for treatment and control arms for a single study. However, if they are different, say, the arm with a longer duration has more adverse events, harmful effects of the study would be seriously biased due to the unbalance of the treatment duration; further, this bias would “contaminate” the results of a meta-analysis. Unfortunately, the potential difference of the treatment duration between the arms was mostly neglected—we only recorded 2 systematic reviews that addressed this problem by using person-time. Therefore, we advocated researchers to consider both the difference in treatment duration between the arms as well as among the studies as part of their analysis and interpretation of results.

Dosage is another important modifier for harmful effects [25, 26]. An increasing number of clinical guidelines have highlighted the importance of establishing a dose–response relationship between intervention and outcomes [27–30]. In network meta-analysis, some authors treated the same drug with different dosages as different drugs, allowing them to model the dosage in the meta-analytic model as a solution to investigating the potential dose–response effects [31, 32]. For the pairwise meta-analysis, subgroup analysis and meta-regression meta-analysis are straightforward; we can model dosage into the model through a mixed linear model or other one-stage methods [26, 33]. One important point is how dosage was quantified. There were two methods, i.e., estimating the dose for each intake and the total dose during the entire treatment. The latter accounts for the treatment duration, but the former did not. There is currently no consensus about which method is better for investigating the dose–response relationship [34]. Moreover, for some topics such as cancer, an intervention may involve two or more drugs for combination therapy. In such cases, it is difficult to measure the dose for the analysis. Further research on these issues would be of interest.

For the risk of bias, one important bias is the selective non-reporting bias for harm outcomes. It refers to the bias that researchers tend to underreport the adverse events in their trials to avoid the potential negative impact on the study findings [35]. The occurrence of reporting bias would push harmful effects into the null and then bias the results. It is estimated that about 50% of the randomized controlled trials inadequately reported the clinical adverse events [36]. Another important bias might be the lack of blinding as well as the funding bias. Previous researchers have shown that trials that lack blinding or received industry funding would exaggerate the treatment effects [37]. However, whether they impacted harmful effects is unclear, and our ongoing parallel project (see Additional file 1) will address this issue. As it can be seen from this study, for the three important sources of bias, very few systematic reviews investigated their potential impacts on harmful effects.

To the best of our knowledge, this is the first study that highlights the importance of investigating the potential impact of effect modifiers on harmful effects. In this study, we presented 6 types of effect modifiers closely related to harmful effects. It is recommended that, for future systematic reviews of adverse events, these six effect modifiers should be routinely considered. Based on the findings of this study, perhaps a domain of “addressing effect modifiers” should be considered in the guideline for meta-analyses of adverse events.

Several limitations should be highlighted. First, in our dataset, we only recorded four Cochrane reviews. This is because most Cochrane reviews generally investigated both efficacy and safety outcomes and therefore did not meet the inclusion criteria of this study. The results of this study may not be representative of Cochrane reviews. Second, for the participants domain of the modifiers, we only consider age as a representative while failing to consider other characteristics (e.g., gender, medical condition, the severity of illness) due to the limited information reported by systematic review authors. This does not mean other characters are not important, in contrast, they are also important and should be considered in light of the real conditions. In addition, this study may also be at risk of reporting bias by systematic reviews — the collected information largely relies on how these systematic reviews were reported. In some cases, systematic reviews have investigated the impact of the effect modifiers on harmful effects while they did not report it. Therefore, the proportions in this study might be underestimated. Moreover, we only considered the impact of these effect modifiers on harmful effects, but not on the baseline risks from meta-analyses of incidence proportions. It is reasonable that these effect modifiers could also impact the baseline risks of the adverse events, and future meta-analyses of incidence proportions should also consider the impacts of potential risk modifiers.

In conclusion, based on empirical evidence, current systematic reviews rarely investigated the potential impact of the important effect modifiers on harmful effects. The development of a review protocol may be helpful to improve this worrisome situation. In addition, further methodological guidelines for meta-analysis of adverse events should consider “effect modifier” as one of the domains to help systematic review authors better investigate harmful effects.

## Supplementary Information

**Additional file 1.**  Protocol

**Additional file 2: Figure S1.** Flow plot

**Additional file 3: Table S1.** Investigation of the impact of effect modifiers on harmful effects in eligible systematic reviews (*N*=279)

**Additional file 4: Table S2.** Investigation of harmful effects in pair-wise meta-analyses (*N*=241)

**Additional file 5: Table S3.** Investigation of harmful effects in pair-wise meta-analyses with the maximum number of studies across outcomes more than or equal to 5 (*N*=208) or 10 (*N*=131)

**Additional file 6.** Included studies and extracted raw data

## Data Availability

All data generated or analysed during this study are included in this published article and extracted raw data could be obtained through supplementary information file (Additional file 6).
