# Supplementary material for: Effect of important modifiers on harmful effects in evidence synthesis practice of adverse events were insufficiently investigated: an empirical investigation
Source: BMC Med Res Methodol. 2023 Apr 28;23:106. doi: 10.1186/s12874-023-01928-2 (PMC10142201; doi:10.1186/s12874-023-01928-2)
Supplement: Supplementary file 1 — Additional file 1. Protocol [file 12874_2023_1928_MOESM1_ESM.docx]

## Supplementary material

## Additional file 1

## Protocol (11-April, 2021)

## Empirical investigation of potential bias for the harmful effects in randomized controlled trials

Chang Xu^1, 2^, Liliane Zorzela^3^, Luis Furuya-Kanamori^4^, Lifeng Lin^5^, Jiaxin Zhang^6^, Sunita Vohra^3,7^

1. Department of Population Medicine, College of Medicine, Qatar University, Al Jamiaa Street, P. O. Box 2713, Doha, Qatar;
2. Chinese Evidence-based Medicine Center, West China Hospital, Sichuan University, Chengdu, China;
3. Department of Pediatrics, Faculty of Medicine & Dentistry, University of Alberta, Edmonton, Alberta, Canada
4. UQ Centre for Clinical Research, Faculty of Medicine, University of Queensland, Brisbane, Australia
5. Department of Statistics, Florida State University, Tallahassee, FL, USA
6. Department of Pharmacy, Guizhou Provincial People's Hospital, Guiyang, China
7. Department of Psychiatry, Faculty of Medicine & Dentistry, University of Alberta, Edmonton, Alberta, Canada

## ^🖂^Correspondence to:

Dr. Chang Xu, Department of Population Medicine, College of Medicine, Qatar University, Al Jamiaa Street, P. O. Box 2713, Doha, Qatar; Email: [xuchang2016@runbox.com](mailto:xuchang2016@runbox.com)

## Research questions

Safety is as important as effectiveness in the assessment of health interventions. As highlighted by the latest Cochrane handbook (version 6.2), all systematic reviews of interventions should investigate the adverse effects of the intervention.^1^ Rare adverse events pose a substantial challenge for statistical modeling and inference for safety assessment, for example, low statistical power, biased effect estimation.^2^ There are many potential factors that cause events to be rare in single trials. These factors result in either random error or systematic error that may bias the estimation of treatment effect. For example, low incidence, small sample size, and limited treatment duration may lead to large random errors; ^3-6^ while selective non-reporting or industry funding may lead to large systematic errors that bias estimates of treatment effects.^7-10^

One example can illustrate the above bias clearly. Suppose the true incidence rate (per month) of a certain adverse event (AE) are 0.0005 and 0.0001 for treatment A and treatment B, respectively. The expected risk ratio (RR) (A vs. B) is therefore 0.0005/0.0001=5. Now let’s suppose a randomized controlled trial (RCT), with the sample sizes are 200 vs. 200, the treatment duration time is 1 month and therefore the expected events in both arms are 200*0.0005*1=0.1, 200*0.0001*1=0.02 and then the observed events could be both 0. Thus, the estimated “pseudo” RR is then 1 that is largely biased. However, if we extend the treatment duration time to 12 months, then the expected events are 0.1*12=1.2, 0.02*12=0.024 and therefore the observed events could be 1 and 0, with an estimated RR of 3 that much closer to true effect. Now if researchers do not report the 1 event as they think this may unfavorable for their new drug, the “adjusted” RR then back to 1 and showed no difference of the risk.

Considering the substantial impact of these factors on the estimate of treatment effects and conclusions of safety assessment, we plan to employ a large-scale empirical investigation to see the extent of the impact and seek potential solutions to adjust the bias by these factors.

## Methods

***Database***

We will use the database of our recent ongoing project that investigated the preference of systematic review authors in dealing with studies with no events in meta-analyses of adverse events. The database collected systematic reviews of healthcare interventions with safety as an exclusive outcome indexed in PubMed from 1-Jan, 2015 to 1-Jan, 2020.

***Eligibility***

The following systematic reviews will be included:

1. Safety as the exclusive outcome;
2. Includes only clinical trials;
3. Contains meta-analysis which with at least 5 studies;
4. Any drug or biological agent as intervention, with comparison of any active or non-active drugs drug, biological agent, or placebo;
5. Provided 2 by 2 table data for each study in the table or forest plot;

We plan to limit meta-analyses for at least 5 studies for two reasons. First, the comparison (e.g. for-profit funding vs. not-for-profit funding) will be based on matching (e.g. sample size, treatment duration, incidence), more studies in a meta-analysis mean a higher possibility for successful matching; Second, our simulation suggested that a meta-analysis with less than 5 studies is inconclusive.

We plan to limit intervention to drugs and biological agents since they are more likely to be funded by industry. Our requirement that original 2 by 2 table data for each study be provided is based on the consideration that most of the meta-analyses did not appropriately deal with zero-events studies, leading to further systematic error. Such systematic error will bias our comparison.

## Context

This study will focus on the potential impact of incidence, sample size, treatment duration time, non-reporting bias, and source of funding on harms reporting in trials, with a special focus on treatment duration time and source of funding. This will be conducted by comparing harms reported in trials with, for example, long treatment duration time/industry funding, to those with short treatment duration time/non- industry funding. Considering the potential impact of the other remaining factors (e.g. sample size, non-reporting bias), we plan to using the matching method whenever possible, say, when comparing the effects of industry funded trials with non-profit institution funded trials, we will match the trials by baseline incidence, sample size, treatment duration, risk of bias.

Therefore, thus information (i.e. 2 by 2 table, treatment duration time, risk of bias, source of funding) will be extracted for each trial in each eligible systematic review. In addition, baseline information like year of publication, original data for meta-analysis, related topics (e.g. cancer) will be also extracted.

## Additional analysis

Meta-regression analysis for those eligible meta-analyses with 10 or more studies will be conducted for treatment duration time (or other factors is possible).

## Main outcome(s)

The main outcome is the relative odds ratio (ROR) of each matched pair trials. In addition, the topic-specific pooled ROR based on inverse variance heterogeneous (IVhet) model will be calculated.^11^ Considering that the method for dealing with zero-events may impact the pair-specific ROR and further impact the pooled ROR, we only consider trials with 1:1 design or close to 1: 1 (i.e., ratio ranges from 0.51 to 1.99). Because under 1:1 design, the continuity correction (add 0.5) works well for both single-arm-zero-events and double-arm-zero-events.

## Secondary outcome(s)

- The proportion of single-arm-zero-events studies and double-arm-zero-events studies within a meta-analysis.
- The potential impact of treatment duration time, non-reporting bias, source of funding for the occurrence of zero-events.

The following information will also be of interest for each eligible meta-analysis that reflect to what extend the harmful effects were investigated:

1. Whether the meta-analysis investigated the potential impact of different treatment/control on the harmful effects?
2. Whether the meta-analysis investigated the potential impact of treatment duration on the harmful effects?
3. Whether the meta-analysis investigated the potential impact of doses of drug on the harmful effects?
4. Whether the meta-analysis investigated the potential impact of funding source on the harmful effects?
5. Whether the meta-analysis investigated the potential impact of risk of bias on the harmful effects?
6. Whether the meta-analysis investigated the potential impact of age on the harmful effects?
7. Whether the meta-analysis rank the confidence of the evidence of harm effects?

## Data extraction (selection and coding)

Data extraction will be done by the lead author, and further checked by a research assistant. See “context” section for information to be exacted.

## Analysis of subgroups or subsets

Year of publication of trials, sample size, incidence.

## Contact details for further information

[xuchang2016@runbox.com](mailto:xuchang2016@runbox.com)

## Conflicts of interest

We declare no conflict of interest.

## Funding

This study did not receive any financial supporting.

**Stage of review**

Review Ongoing: Literature screen for current study has not been done (By 11^th^-April, 2021).

**Records of deviations of protocol and the article**

| 11-April, 2021 | Draft the protocol |
| --- | --- |
| 14-April, 2021 | Changing “follow-up time” to “treatment time” after consulting experts of clinical trials.  **Reason:** Any treatment has a wash-out period, after the period, any adverse events may not be caused by intervention. |
| 17-April, 2021 | Send for collaborators for reviewing, no context changes |
| 22-April, 2021 | Distinguishing the treatment duration and control duration in data extraction form based on extraction training |
| 25-April, 2021 | Adding more information in secondary outcome, say, the seven “Whether…”, as an effort to see how well the harm effects were investigated |
| 4-May, 2021 | Change inclusion criteria: meta-analysis of RCTs to meta-analysis of clinical trials.  Reason: Many systematic reviews claimed they only included RCTs, while some of these “RCTs” were not RCT, for example, only have one-arm, and not used for the meta-analysis of harmful effects. |
| 15-May, 2021 | During the data extraction, we noticed many of the systematic reviews failed to report or conduct the risk of bias of each trial. After an online meeting, we decided to assess the risk of bias by our research team. |
| 17-May, 2021 | Two assistants added in the research, with background of evidence-based medicine. They take charges of the assessment of risk of bias. Since the assessment of ROB is of some subjective, the two assistants are blinded, they were not and will not inform the aim of the project and the potential comparisons. And the assessment of risk of bias will be started after the finish of the data extraction (possibly in July). |
| 28-May, 2021 | Change in analysis of the main outcomes: We primarily plan to limit RCTs with 1: 1 design to facilitate the estimation of the ORs in the case of zero-events. But then after our recent simulation study, we found continuity correction works well even when the ratio ranges from 0.51 to 1.99, therefore we “relaxed” the limits to 0.51 to 1.99. |
| 1-June, 2021 | We primarily collected data of the clinical trials from the systematic reviews, while for the sample we finished (accounted for 1/3 of the total), we found that many of the data were incorrectly recorded by these systematic reviews. Based on an urgent online meeting (CX and TQ), we decided to re-do the data extraction, directly from the original studies. |
| 5-June, 2021 | Online meeting with a pharmacist about the definition of different treatments/controls. |
| 10-June, 2021 | Some clinical trials may have a flexible treatment schedule, for example, they use A drug with 5 mg at week 1, and 10 mg at week 2, and 20 mg at week 3, and then continue 20 mg for the following 11 weeks.  Makes it difficult to extract the dose. After a discussion (CX and TQ), we decided to use the maximum dose in our study. |

**Reference.**

1. Peryer G, Golder S, Junqueira DR, Vohra S, Loke YK; on behalf of the Cochrane Adverse Effects Methods Group. Chapter 19: Adverse effects. Cochrane Handbook for Systematic Reviews of Interventions version 6.2 (updated February 2021). *Cochrane,* 2021. Available from [www.training.cochrane.org/handbook](http://www.training.cochrane.org/handbook).
2. Jia P, Lin L, Kwong JSW, Xu C. Many meta-analyses of rare events in the Cochrane Database of Systematic Reviews were underpowered. *J Clin Epidemiol.* 2021;131:113-122.
3. Xu C, Li L, Lin L, et al. Exclusion of studies with no events in both arms in meta-analysis impacted the conclusions. *J Clin Epidemiol.* 2020;123:91-99.
4. Ju K, Lin L, Chu H, Cheng LL, Xu C. Laplace approximation, penalized quasi-likelihood, and adaptive Gauss-Hermite quadrature for generalized linear mixed models: towards meta-analysis of binary outcome with sparse data. *BMC Med Res Methodol.* 2020;20(1):152.
5. Xu C, Furuya-Kanamori L, Zorzela L, Lin L, Vohra S. A proposed framework to guide evidence synthesis practice for meta-analysis with zero-events studies. *J Clin Epidemiol.* 2021; 135:70-78.
6. Zorzela L, Loke YK, Ioannidis JP, et al. PRISMA harms checklist: improving harms reporting in systematic reviews. *BMJ.* 2016;352: i157.
7. Aylin P, Tanna S, Bottle A, Jarman B. How often are adverse events reported in English hospital statistics? *BMJ.* 2004; 329(7462):369.
8. Janiaud P, Cristea IA, Ioannidis JPA. Industry-funded versus non-profit-funded critical care research: a meta-epidemiological overview. *Intensive Care Med.* 2018; 44(10):1613-1627.
9. Ioannidis JP, Lau J. Completeness of safety reporting in randomized trials: an evaluation of 7 medical areas. *JAMA.* 2001;285(4):437-443.
10. Als-Nielsen B, Chen W, Gluud C, Kjaergard LL. Association of funding and conclusions in randomized drug trials: a reflection of treatment effect or adverse events? *JAMA.* 2003; 290(7):921-928.
11. Doi SA, Barendregt JJ, Khan S, Thalib L, Williams GM. Advances in the meta-analysis of heterogeneous clinical trials I: The inverse variance heterogeneity model. *Contemp Clin Trials.* 2015;45(Pt A):130-138.
