# Supplementary figures and images for: Effect of important modifiers on harmful effects in evidence synthesis practice of adverse events were insufficiently investigated: an empirical investigation
Source: BMC Med Res Methodol. 2023 Apr 28;23:106. doi: 10.1186/s12874-023-01928-2 (PMC10142201; doi:10.1186/s12874-023-01928-2)

## Additional file 2

**
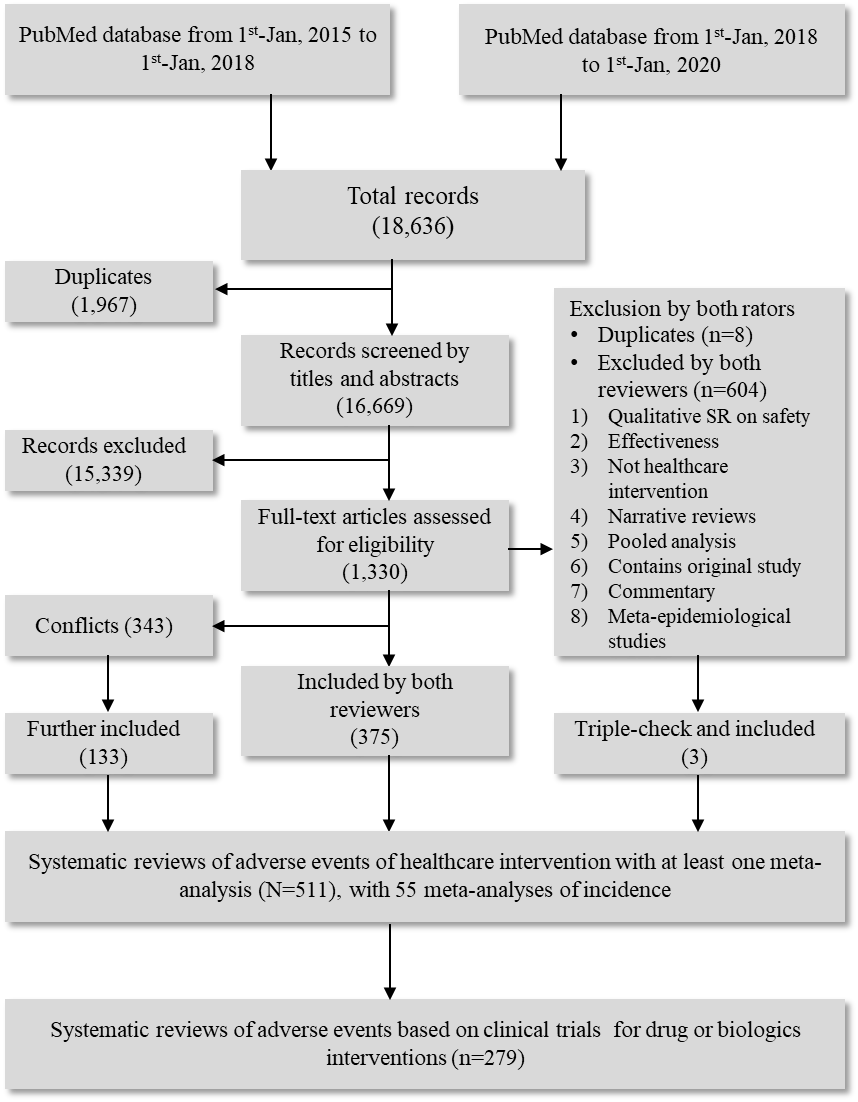
**

**Figure 1.** Flow plot

Supplement: Supplementary file 2 — Additional file 2: Figure S1. Flow plot [file 12874_2023_1928_MOESM2_ESM.docx]
