# Supplementary material for: Effect of important modifiers on harmful effects in evidence synthesis practice of adverse events were insufficiently investigated: an empirical investigation
Source: BMC Med Res Methodol. 2023 Apr 28;23:106. doi: 10.1186/s12874-023-01928-2 (PMC10142201; doi:10.1186/s12874-023-01928-2)
Supplement: Supplementary file 3 — Additional file 3: Table S1. Investigation of the impact of effect modifiers on harmful effects in eligible systematic reviews (N=279) [file 12874_2023_1928_MOESM3_ESM.docx]

## Additional file 3

**Table 1.** **Investigation of the impact of effect modifiers on harmful effects in eligible systematic reviews (n=279)**

| **Investigation of harmful effects** | **Yes (%)** | **No (%)** | **NA (%)** |
| --- | --- | --- | --- |
| 1. Whether the authors investigated the potential impact of different interventions/controls on harmful effects in meta-analysis? | 197 (70.61%) | 43 (15.41%) | 39 (13.98%) |
| 1. Whether the authors investigated the potential impact of treatment duration on harmful effects in meta-analysis? | 59 (21.15%) | 216 (77.42%) | 4 (1.43%) |
| 1. Whether the authors investigated the potential impact of drug doses on harmful effects in meta-analysis? | 69 (24.73%) | 206 (73.84%) | 4 (1.43%) |
| 1. Whether the authors investigated the potential impact of age on harmful effects in meta-analysis? | 32 (11.47%) | 247 (88.53%) | 0 |
| 1. Whether the authors investigated the potential impact of risk of bias on harmful effects in meta-analysis? | 18 (6.45%) | 260 (93.19%) | 1 (0.36%) |
| - Allocation concealment | 2 (0.72%) | 276 (98.92%) | 1 (0.36%) |
| - Random sequence generation | 2 (0.72%) | 276 (98.92%) | 1 (0.36%) |
| - Blinding of participants and personnel/outcome assessment | 9 (3.23%) | 269 (96.42%) | 1 (0.36%) |
| - Selective reporting | 2 (0.72%) | 276 (98.92%) | 1 (0.36%) |
| - By overall risk of bias | 8 (2.87%) | 270 (96.77%) | 1 (0.36%) |
| 1. Whether the authors investigated the potential impact of the source of funding on harmful effects in meta-analysis? | 3 (1.08%) | 275 (98.57%) | 1 (0.36%) |
| 1. Whether the authors rank the confidence of the evidence of harmful effects? | 31 (11.11%) | 248 (88.89%) | 0 |

NA: not applicable, when all included studies have the same treatment/control, or the same treatment duration, or same age, or same risk of bias, or same funding source.
