# Supplementary material for: Effect of important modifiers on harmful effects in evidence synthesis practice of adverse events were insufficiently investigated: an empirical investigation
Source: BMC Med Res Methodol. 2023 Apr 28;23:106. doi: 10.1186/s12874-023-01928-2 (PMC10142201; doi:10.1186/s12874-023-01928-2)
Supplement: Supplementary file 4 — Additional file 4: Table S2. Investigation of harmful effects in pair-wise meta-analyses (N=241) [file 12874_2023_1928_MOESM4_ESM.docx]

## Additional file 4

**Table 2. Investigation of harmful effects in pair-wise meta-analyses (N=241)**

| **Investigation of harmful effects** | **Yes (%)** | **No (%)** | **NA (%)** |
| --- | --- | --- | --- |
| 1. Whether the authors investigated the potential impact of different intervention/control on the harmful effects in meta-analysis? | 159 (65.98%) | 43 (17.84%) | 39 (16.18%) |
| 1. Whether the authors investigated the potential impact of treatment duration on the harmful effects in meta-analysis? | 46 (19.09%) | 192 (79.67%) | 3 (1.24%) |
| 1. Whether the authors investigated the potential impact of doses of drug on the harmful effects in meta-analysis? | 63 (26.14%) | 174 (72.20%) | 4 (1.66%) |
| 1. Whether the authors investigated the potential impact of age on the harmful effects in meta-analysis? | 26 (10.79%) | 215 (89.21%) | 0 |
| 1. Whether the authors investigated the potential impact of risk of bias on the harmful effects in meta-analysis? | 16 (6.64%) | 224 (92.95%) | 1 (0.41%) |
| - Allocation concealment | 2 (0.83%) | 238 (98.76%) | 1 (0.41%) |
| - Random sequence generation | 2 (0.83%) | 238 (98.76%) | 1 (0.41%) |
| - Blinding of participants and personnel/ outcome assessment | 9 (3.73%) | 231 (95.85%) | 1 (0.41%) |
| - Selective reporting | 2 (0.83%) | 238 (98.76%) | 1 (0.41%) |
| - By overall risk of bias | 6 (2.49%) | 234 (97.10%) | 1 (0.41%) |
| 1. Whether authors investigated the potential impact of source of funding on the harmful effects in meta-analysis? | 3 (1.24%) | 238(98.76%) | 0 |
| 1. Whether the authors rank the confidence of the evidence of harm effects? | 27 (11.20%) | 214 (88.80%) | 0 |

NA: not applicable, when all included studies have the same treatment/control, or the same treatment duration, or same age, or same risk of bias, or same funding source.
