# Supplementary material for: Effect of important modifiers on harmful effects in evidence synthesis practice of adverse events were insufficiently investigated: an empirical investigation
Source: BMC Med Res Methodol. 2023 Apr 28;23:106. doi: 10.1186/s12874-023-01928-2 (PMC10142201; doi:10.1186/s12874-023-01928-2)
Supplement: Supplementary file 5 — Additional file 5: Table S3. Investigation of harmful effects in pair-wise meta-analyses with the maximum number of studies across outcomes more than or equal to 5 (N=208) or 10 (N=131) [file 12874_2023_1928_MOESM5_ESM.docx]

## Additional file 5

**Table 3. Investigation of harmful effects in pair-wise meta-analyses with the maximum number of studies across outcomes more than or equal to 5 (N=208) or 10 (N=131)**

| **Investigation of harmful effects** | **the maximum number of studies across outcomes more than or equal to 5 (N=208)** | | | **the maximum number of studies across outcomes more than or equal to 10 (N=131)** | | |
| --- | --- | --- | --- | --- | --- | --- |
|  | **Yes (%)** | **No (%)** | **NA (%)** | **Yes (%)** | **No (%)** | **NA (%)** |
| 1. Whether the authors investigated the potential impact of different intervention /control on the harmful effects in meta-analysis? | 145 (69.71%) | 34 (16.35%) | 29 (13.94%) | 95 (72.52%) | 17 (12.98%) | 19 (14.50%) |
| 1. Whether the authors investigated the potential impact of treatment duration on the harmful effects in meta-analysis? | 44 (21.15%) | 163 (78.37%) | 1 (0.48%) | 38 (29.01%) | 92 (70.23%) | 1 (0.76%) |
| 1. Whether the authors investigated the potential impact of doses of drug on the harmful effects in meta-analysis? | 57 (27.40%) | 148 (71.15%) | 3 (1.44%) | 44 (33.59%) | 85 (64.89%) | 2 (1.53%) |
| 1. Whether the authors investigated the potential impact of age on the harmful effects in meta-analysis? | 25 (12.02%) | 183 (87.98%) | 0 | 22 (16.79%) | 109 (83.21%) | 0 |
| 1. Whether the authors investigated the potential impact of risk of bias on the harmful effects in meta-analysis? | 15 (7.21%) | 193 (92.79%) | 0 | 12 (9.16%) | 119 (90.84%) | 0 |
| - Allocation concealment | 2 (0.96%) | 206 (99.04%) | 0 | 1 (0.76%) | 130 (99.24%) | 0 |
| - Random sequence generation | 2 (0.96%) | 206 (99.04%) | 0 | 1 (0.76%) | 130 (99.24%) | 0 |
| - Blinding of participants and personnel/ outcome assessment | 8 (3.85%) | 200 (96.15%) | 0 | 6 (4.58%) | 125 (95.42%) | 0 |
| - Selective reporting | 2 (0.96%) | 206 (99.04%) | 0 | 1 (0.76%) | 130 (99.24%) | 0 |
| - By overall risk of bias | 6 (2.88%) | 202 (97.12%) | 0 | 5 (3.82%) | 126 (96.18%) | 0 |
| 1. Whether authors investigated the potential impact of source of funding on the harmful effects in meta-analysis? | 3 (1.44%) | 205 (98.56%) | 0 | 3 (2.29%) | 128 (97.71%) | 0 |
| 1. Whether authors rank the confidence of the evidence of harm effects? | 26 (12.50%) | 182 (87.50%) | 0 | 22 (16.79%) | 109 (83.21%) | 0 |

NA: not applicable, when all included studies have the same treatment/control, or the same treatment duration, or same age, or same risk of bias, or same funding source.
